# Laryngeal Manifestation of Nodular Fasciitis: A Case Report and Literature Review

**DOI:** 10.7759/cureus.19836

**Published:** 2021-11-23

**Authors:** Rupert Stadlhofer, Andreas Lübke, Arne Böttcher

**Affiliations:** 1 Otorhinolaryngology, University Medical Center Hamburg-Eppendorf, Hamburg, DEU; 2 Pathology, University Medical Center Hamburg-Eppendorf, Hamburg, DEU

**Keywords:** nodular fasciitis, head and neck, neoplasm, benign laryngeal tumor, laryngology

## Abstract

Nodular fasciitis (NF) are non-neoplastic, fibroblastic lesions, typically located on the trunk and the extremities. The incidence of NF in the head and neck region is 13%-20%. However, a manifestation in the larynx of adult patients is extremely rare. Therefore, the occurrence of NF in this region can lead to diagnostic challenges and a high risk of misdiagnosis as well as potential mishandling when not aware of its possible laryngeal manifestation.

Following emergency admission of a 41-year-old woman to the emergency department (ED) due to progressive dyspnea and inspiratory stridor a transnasal flexible laryngeal endoscopic examination revealed a left-lateral, subglottic mass. A subsequently performed CT demonstrated a 2.2 cm x 1.5 cm sized lesion of the subglottic larynx with profound stenosis of the lumen (Myer-Cotton grade III), no extraluminal extension, and no distant metastases. Histopathological processing of a tissue sample obtained by microlaryngoscopy and translaryngeal tracheoscopy revealed a spindle-cell lesion with immunohistochemical and molecular-pathogenic profile of NF. After tumor debulking and steroid infiltration (triamcinolone), a regrowth tendency quickly became apparent, which is why a tracheostomy had to be performed. Laryngectomy was rejected by the patient. After multiple transoral tumor reduction attempts, radiotherapy was performed according to an interdisciplinary tumor board decision to limit regrowth tendency. Subsequently, a substantial reduction of the tumor volume could be seen, although a discreet stenosis of the subglottic tracheal lumen persists in the follow-up.

Laryngeal NF poses several challenges due to its rare occurrence in this location. This case report emphasizes the knowledge of this differential diagnosis and also depicts an interdisciplinary therapeutic approach aiming for function-preserving treatment of this benign but potentially relapsing pathology.

## Introduction

Nodular fasciitis (NF) is a benign, myofibroblastic, self-limiting mesenchymal neoplasm that typically develops on the surface of the fascia and extends into the subcutis. This neoplasm can occur in any location; the upper extremity, trunk, and head and neck are the most prevalent sites. Up to 25% of all NF cases are located in the head and neck region [1]. However, a manifestation in the larynx of adult patients is extremely rare and was only described twice in literature. Therefore, the occurrence of NF in this region can lead to diagnostic challenges and a high risk of misdiagnosis as well as potential mishandling when not aware of its possible laryngeal manifestation. 

## Case presentation

**Figure 1:**
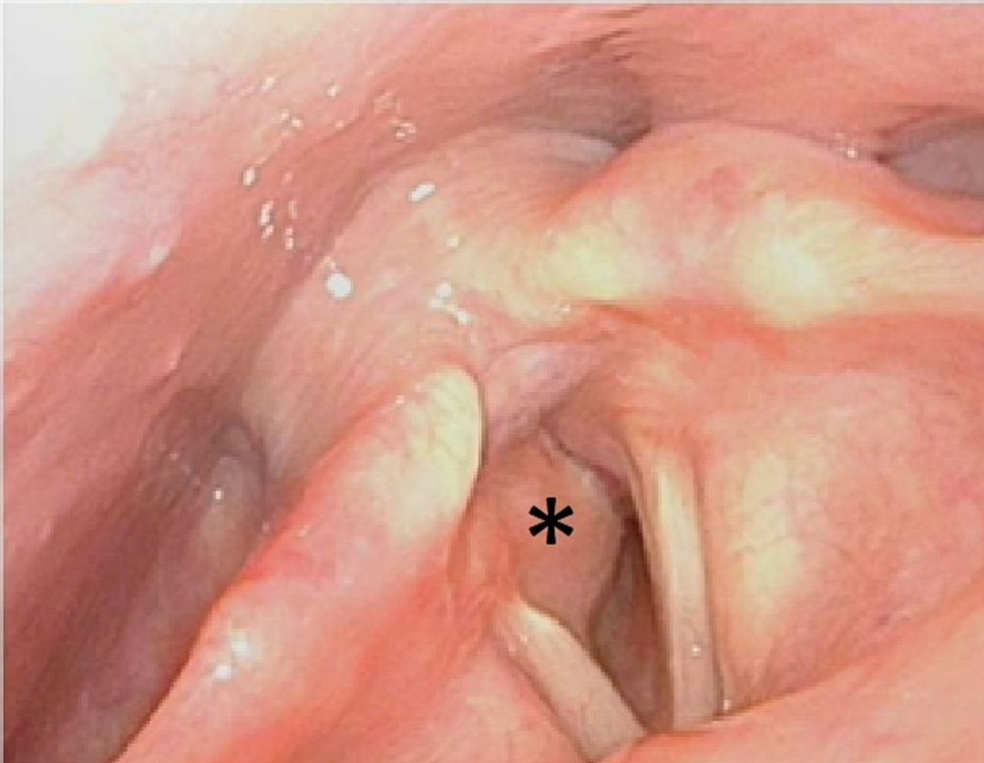
Subglottic mass (*) shown via preoperative transnasal endoscopy.

**Figure 2:**
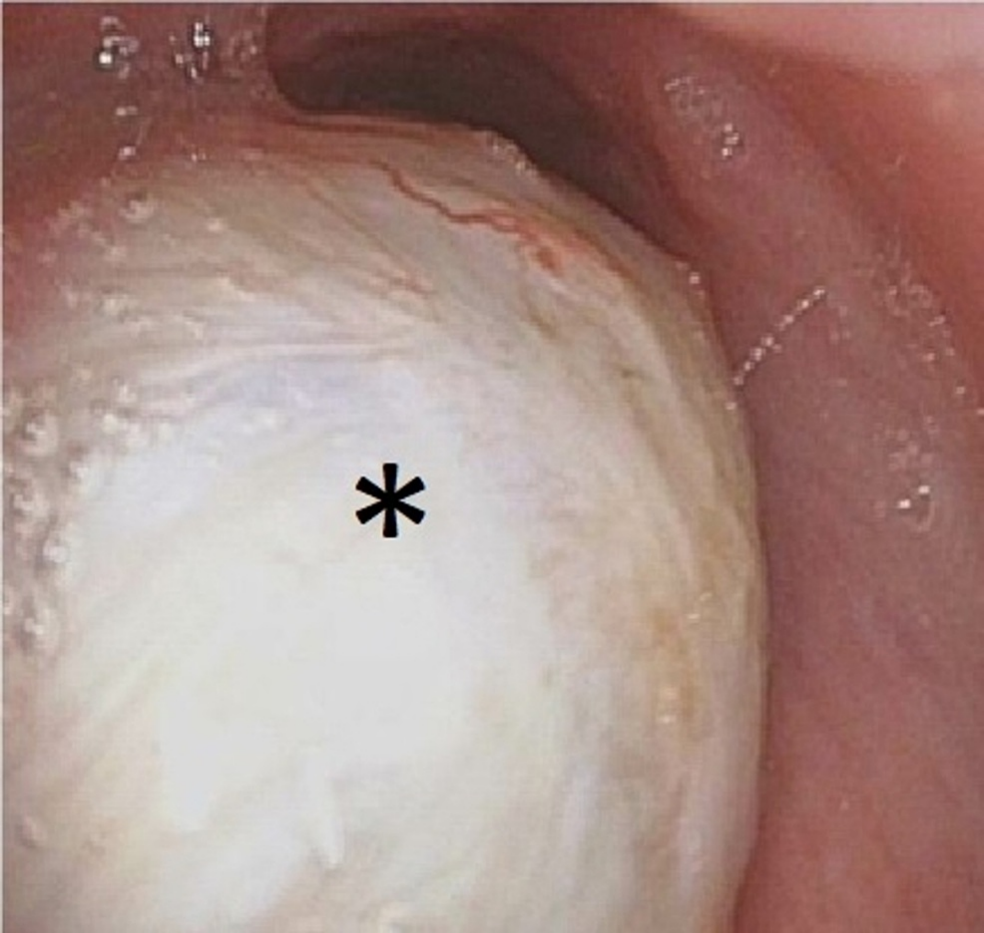
Subglottic mass (*) shown via intraoperative transstomal upwards endoscopy.

A 41-year-old woman with a medical history of hypertension, gastroesophageal reflux, and obesity (BMI: 50.1 kg/m2) was transferred to the emergency department (ED) due to progressive dyspnea and inspiratory stridor over the last three days. The patient showed no dysphagia or dysphonia. A transnasal flexible laryngeal endoscopic examination revealed a right-lateral, subglottic mass (Figures 1-2).

**Figure 3:**
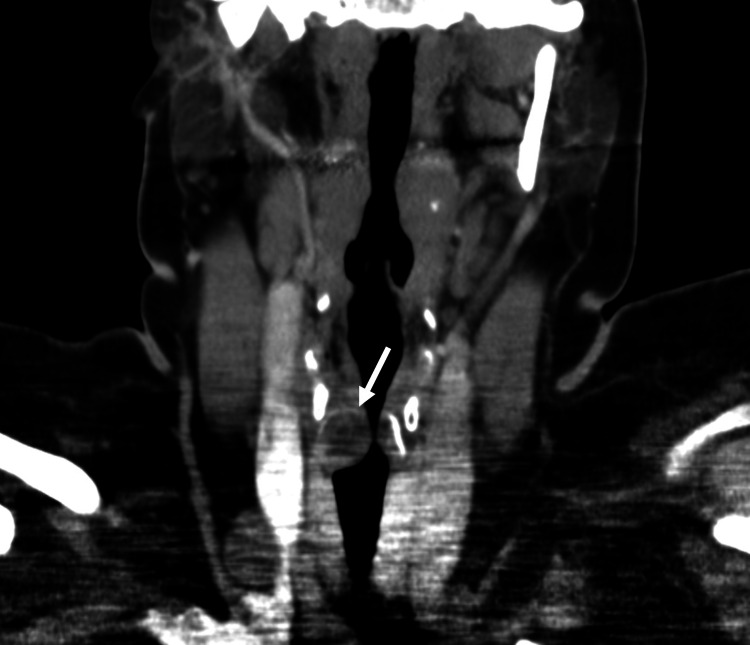
CT scan showing right-sided subglottic mass (2.2 cm x 1.5 cm) (white arrow).

No further laryngeal malformation was detectable. A subsequently performed CT of the neck, thorax, and abdomen demonstrated a 2.2 cm x 1.5 cm sized lesion with well-defined margins located in the right-sided, subglottic larynx with profound stenosis (Myer-Cotton grade III) of the lumen but no extraluminal extension or distant metastases (Figure 3).

**Figure 4:**
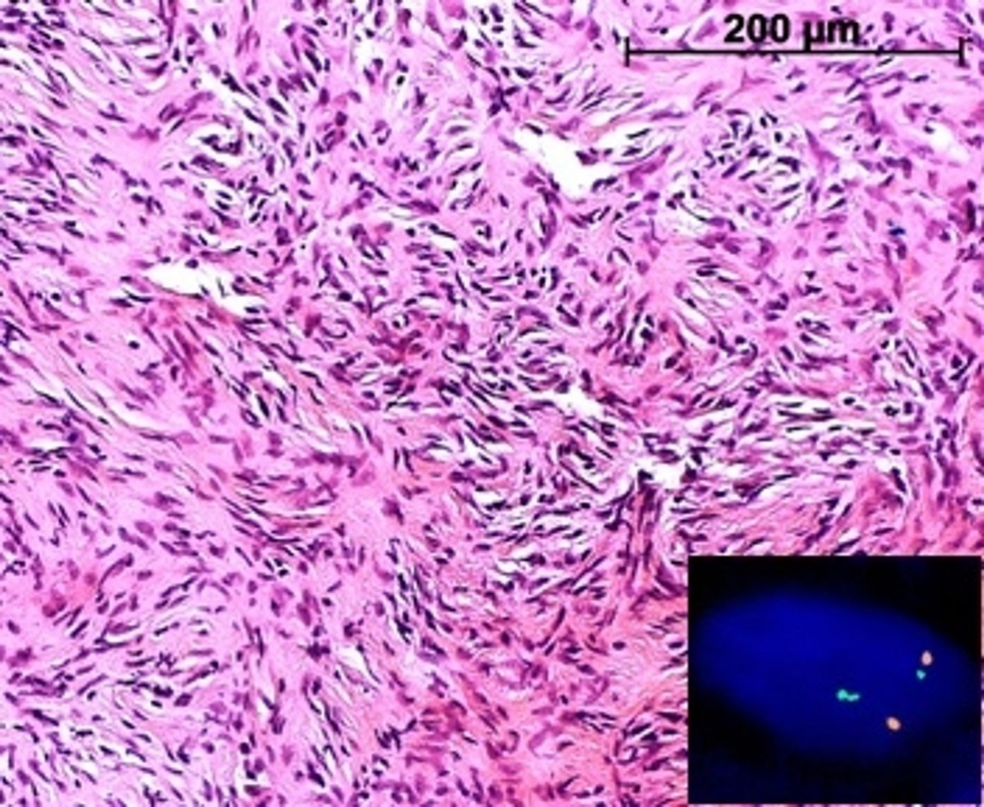
Hematoxylin and eosin stain of lesional cells (magnification factor 200); spindle cells without significant cellular atypia with the aspect of a cell culture. Inset: FISH showing USP6 translocation in the lesional cells. FISH, fluorescence in situ hybridization; USP6, ubiquitin-specific protease 6

Tissue samples were obtained by microlaryngoscopy and translaryngeal tracheoscopy in jet ventilation. Histology revealed a moderately cellular myofibroblastic lesion with a noticeable myxoid matrix. The tumor cells had tapering or ovoid nuclei without any atypia or pleomorphism and indistinct palely eosinophilic cytoplasm. Multifocally, prominent osteoclastic giant cells were present, and the myxoid stroma contained scattered chronic inflammatory cells. Immunohistochemistry showed uncharacteristic partial positivity for smooth muscle actin (SMA) and negative staining results for AE1/AE3, desmin, S100, CD34, STAT6, and EMA. The ki-67 proliferative index was 3%. Fluorescence in situ hybridization (Figure 4) identified a USP6 (ubiquitin-specific protease 6) gene rearrangement. In summary, the findings fit well with NF.

After tumor debulking and steroid infiltration (triamcinolone; 10%), a regrowth tendency quickly became apparent and due to progressive dyspnea a tracheostomy had to be performed. The patient rejected laryngectomy. After multiple transoral and transstomal tumor resection attempts, postoperative radiotherapy was conducted according to an interdisciplinary tumor and sarcoma board decision to limit regrowth tendency. A conformal photon radiation therapy (6 mV) in the subglottic region was performed with a total of 36.0 gray applicated over 18 sessions. Subsequently, a significant reduction of the tumor volume could be observed after treatment and in the follow-up period. There were sufficient translaryngeal airflow and phonation evident while temporary closure of the tracheostomy, which is why a definite operative closure of the tracheostomy was done 12 months after radiotherapy was completed.

**Figure 5:**
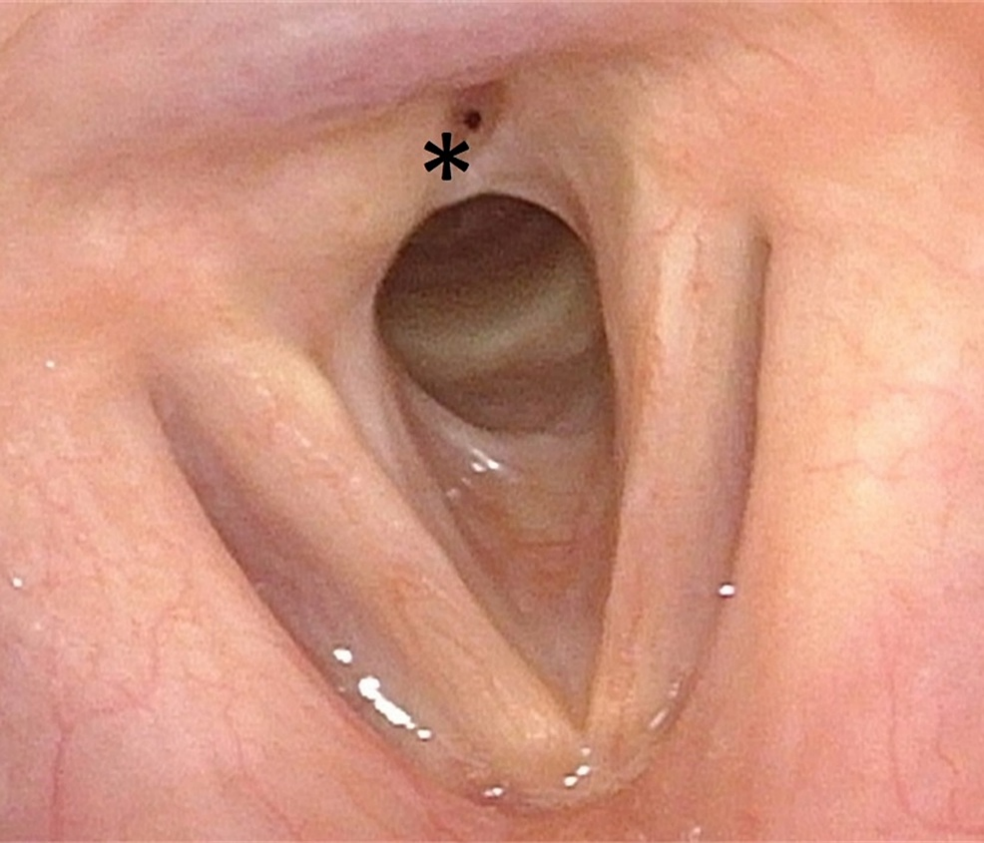
Laryngeal results 16 months after definite treatment showing scar tissue (*) in the dorsal (sub)glottic region via transnasal endoscopy.

Nevertheless, a discrete stenosis of the subglottic lumen persisted due to the formation of sail-shaped scar tissue in the dorsal (sub)glottic region visible in transnasal flexible laryngeal endoscopic examination, which was not showing any signs of functional limitations in spirometry 16 months after definite therapy (Figure 5).

## Discussion

The pathogenesis of NF, which commonly manifests between the second and fourth decade of life, is widely unknown. In many of these lesions, a recurrent USP6 gene rearrangement could be identified, with MYH9 being the most frequent fusion partner (about 70% of cases), characterizing these lesions as benign neoplasms. Due to their self-limiting biological characteristics, the term "transient neoplasia" has been established [2-6].

Surgical removal of NF is the established standard treatment with additional clinical benefit reported for intra- and perilesional corticosteroid injection [7]. Even though partial resection of the lesion showed a relapse rate below 10%, the subsequent growth habit is unpredictable. Following biopsy or subtotal resection, a fast expansion of the tissue volume was observed. This behavior, which was also present in this case, is assumed to be caused by an increased proliferation rate of fibroblasts and myxoid matrix and infiltration of inflammatory cells and neoangiogenesis triggered by surgical manipulation [8].

To the authors' knowledge, there is only one case of laryngeal NF in an adult patient described in literature after its initial description in 1984 by Jones et al. but Al Omari et al. could not provide a histopathologic workup demonstrating a USP6 rearrangement, therefore, being deficient in its diagnostic validity [9-10].

The clinical and histopathological features of these lesions may result in difficulties obtaining the correct diagnosis, often being confused with other more frequent, benign laryngeal entities but also malignancies, such as sarcomas. This may result in the application of inappropriate or even overaggressive treatment strategies. This case report is the first describing a function-preserving treatment strategy for relapsing laryngeal NF.

## Conclusions

Due to the exceptional rare occurrence of NF in the larynx, this case report highlights the diagnostic challenges of a laryngeal manifestation of NF and the importance of its consideration as a differential diagnosis in rapidly growing laryngeal masses. Furthermore, it provides an intradisciplinary treatment approach if growth is not controlled by primary resection and preservation of function is aspired. Long-term patient follow-up is needed due to the potential recurrence of NF.
